# Skeleton-Based Fall Detection with Multiple Inertial Sensors Using Spatial-Temporal Graph Convolutional Networks

**DOI:** 10.3390/s23042153

**Published:** 2023-02-14

**Authors:** Jianjun Yan, Xueqiang Wang, Jiangtao Shi, Shuai Hu

**Affiliations:** 1 Shanghai Key Laboratory of Intelligent Sensing and Detection Technology, East China University of Science and Technology, Shanghai 200237, China; 2 School of Mechanical and Power Engineering, East China University of Science and Technology, Shanghai 200237, China

**Keywords:** fall detection, multiple inertial sensors, skeleton, spatial-temporal graph convolutional networks

## Abstract

The application of wearable devices for fall detection has been the focus of much research over the past few years. One of the most common problems in established fall detection systems is the large number of false positives in the recognition schemes. In this paper, to make full use of the dependence between human joints and improve the accuracy and reliability of fall detection, a fall-recognition method based on the skeleton and spatial-temporal graph convolutional networks (ST-GCN) was proposed, using the human motion data of body joints acquired by inertial measurement units (IMUs). Firstly, the motion data of five inertial sensors were extracted from the UP-Fall dataset and a human skeleton model for fall detection was established through the natural connection relationship of body joints; after that, the ST-GCN-based fall-detection model was established to extract the motion features of human falls and the activities of daily living (ADLs) at the spatial and temporal scales for fall detection; then, the influence of two hyperparameters and window size on the algorithm performance was discussed; finally, the recognition results of ST-GCN were also compared with those of MLP, CNN, RNN, LSTM, TCN, TST, and MiniRocket. The experimental results showed that the ST-GCN fall-detection model outperformed the other seven algorithms in terms of accuracy, precision, recall, and F_1_-score. This study provides a new method for IMU-based fall detection, which has the reference significance for improving the accuracy and robustness of fall detection.

## 1. Introduction

Falling is an unpredictable and irregular human activity. It refers to the process in which a person is suddenly affected by uncontrolled random factors during normal physiological activities, leading to changes in body posture, and finally contact with a low potential object (such as the ground). The physical and cognitive functions of the elderly will deteriorate significantly with age, so when falls occur in the elderly population over the age of 60, falls often cause serious consequences. Falls are the second leading cause of unintentional injury deaths worldwide. Globally, an estimated 684,000 individuals die from falls each year, with more than 80% of these deaths occurring in low-income and middle-income countries. Adults older than 60 years of age suffer the greatest number of fatal falls [1]. Therefore, it is important to develop a reliable and accurate fall-detection system to send fall alert messages to guardians and medical personnel in time to reduce the injury caused by falls to the elderly.

In recent years, research on fall detection has been more and more popular. Domestic and international researchers have conducted many in-depth studies on human activity recognition and fall-detection technologies. In terms of different implementation methods, fall-detection technologies are broadly classified into the following three types: fall-detection technology based on computer vision [2,3,4,5,6], scene sensors [7,8], and wearable devices.

The most important feature of the approach based on computer vision is the simple installation of equipment and intuitive vision. However, it is difficult for a two-dimensional image to separate the subject without a unified background. In addition, the video surveillance equipment can be constrained by the indoor environment, and there are certain monitoring dead spots, and also problems such as leaking personal privacy. The approach based on scene sensors can directly capture human actions without infringing user privacy, but it is difficult to apply in outdoor environments.

The approach based on wearable devices is to wear the sensor devices for collecting human kinematic data on certain parts of the user’s body, and to judge whether a fall has occurred by processing and analyzing the collected human motion data. The development of fall-detection technology based on wearable devices has been very rapid and there are many kinds of devices, most of which are not affected by external factors such as environmental sites and can also detect human movement at any time and any place. In such studies of fall detection, most of the devices used by researchers are based on accelerometers and gyroscopes. They can also protect personal privacy while collecting the users’ movement data. At the same time, with the rapid development of computing chips, wearable devices are becoming lower in cost, smaller in size, and lighter in weight, making them more feasible to promote in real life.

## 2. Related Works

Fall-detection methods based on wearable sensors are currently developing rapidly, and various wearable devices have been emerging, mainly due to their low deployment cost, privacy protection, and lack of time and space constraints. With the development of microelectronics, the size of integrated devices (such as inertial sensors) is getting smaller and smaller, increasing the comfort level of wearable device users. Commonly used fall-detection and daily behavior-classification algorithms can be classified into three main categories: threshold-based methods (TBM), machine learning methods (MLM), and deep learning methods (DLM). TBM has the advantages of computational simplicity and low power consumption. DLM and MLM improve the accuracy and balance the false negatives (undetected falls) and false positives (undetected activities of daily living, ADLs) better.

Hsieh et al. [9] suggested a new hierarchical fall-detection algorithm including threshold-based and knowledge-based approaches to detect fall events. The threshold-based approach efficiently detected fall events from continuous sensor data. The knowledge-based approach used a multistage fall model that included free-fall, impact, and rest phases, which could identify fall events more accurately. Al-Kababji et al. [10] proposed a novel IoT-based fall-detection system that included a sensing device that transmitted data to a mobile application through a gateway device connected to the cloud. Bhattacharjee et al. [11] developed a smart walking assistant (SWA) for elderly care using an intelligent real-time hybrid model, which was capable of identifying whether the faller had recovered within a stipulated period of time. In the case of fall without recovery, an alert message along with date, time, and location of fall would be sent. Sheikh et al. [12] established a wheelchair fall-detection system based on low-cost embedded inertial sensors and unsupervised one-class support vector machines (OCSVM). Nho et al. [13] presented a novel fall-detection method based on the generative adversarial network (GAN) using a heart rate sensor and an accelerometer. Wu et al. [14] constructed a fall-detection system based on wearable sensors. The algorithm used in this system was based on thresholds of sum acceleration and rotation angle information. Hashim et al. [15] designed and implemented a wearable fall-detection system (WFDS) for Parkinson’s Disease (PD) patients based on the low-power ZigBee wireless sensor network (WSN). The falls of PD patients were detected based on the data event algorithm (DEA) results of two wireless sensor nodes, an accelerometer and MyoWare mounted on the patient’s body. Wang et al. [16] proposed a novel cascade and parallel multistate fall-detection algorithm using waist-mounted tri-axial accelerometer signals. The very low computational cost and small size not only enabled it to be embedded in wearable sensors but also required very low system power, which could enhance the autonomy of wearable fall-detection devices.

The threshold-based approaches have low computational complexity and can be easily implemented on wearable devices [17]. Wang et al. [18] developed a fall detector called NEON. NEON used the TBM algorithm to capture the entire fall process, including the falling phase, impact phase and stationary phase. When all action signals matched the characteristics of a fall, NEON would classify the event as a fall and raise an alarm. However, it could only distinguish between a fall and non-fall ADL.

The performance of the MLM and DLM algorithms has been significantly improved compared to the TBM algorithms. Martínez-Villaseñor et al. [19] compared the results of four machine learning models for fall detection: random forest (RF), support vector machines (SVM), multilayer perceptron (MLP), and k-nearest neighbors (kNN). RF and MLP showed the best performance in the experiments of different window sizes, with the highest accuracy of 95.39% and 95.49%, respectively. Delgado-Escano et al. [20] proposed a fall-detection algorithm combining DL and SVM, in which features were extracted by convolutional neural network (CNN) and a classifier of SVM was used to determine whether a fall event had occurred. Torti et al. [21] embedded recursive neural network (RNN) architecture in the micro controller unit (MCU) of a wearable device. García et al. [22] combined a long-short term memory neural network (LSTM) together with a novel data augmentation to classify falls and ADLs. The combination produced a more robust and accurate fall-detection model. Alarifi et al. [23] proposed an effective and optimized fall-detection system that used an approach based on a killer heuristics optimized AlexNet convolution neural network with wearable IoT sensor devices.

However, these normal fall-detection methods based on inertial sensor data have several limitations: (1) the robustness and accuracy of the fall-detection methods based on IMU still need to be improved; (2) the natural connection relationship of human body joints and the structure of the human skeleton are not fully considered. In general, TBM have relatively low accuracy and are unable to balance sensitivity and specificity [24]. TBM, MLM, and DLM are not only unable to express the dependence between joints during the change in human posture, but also cannot effectively extract the spatial-temporal information of human falls and ADLs. To address these issues, we proposed a skeleton-based fall-detection method using spatial-temporal graph convolutional networks with IMUs.

A spatial-temporal graph convolutional network (ST-GCN) [25] is used in the field of video-based human action recognition. As ST-GCN can model dynamic skeletons, a spatial-temporal graph convolutional network based on the human skeleton model was established for fall detection. This network better captures the motion relationship between joints during human action. It also can better mine the action features of human falls and ADLs at spatial and temporal scales through graph spatial convolution and temporal convolution and improve the robustness and accuracy of fall recognition based on IMUs.

The rest of this paper is organized as follows. Section 3 explains the dataset and the proposed ST-GCN-based fall-detection method. Then, Section 4 presents the experiments and results while Section 5 contains the discussion of the results. Finally, conclusion and future works are laid out in Section 6.

## 3. Methods and Data

In this section, the dataset of falls and ADLs is introduced in detail; then, the ST-GCN-based fall-detection method is proposed; finally, the overall structure of the ST-GCN network used in this paper and three partition strategies are clearly described.

To evaluate the performance of the skeleton-based ST-GCN classification model for fall detection, the UP-Fall dataset containing multiple inertial sensors data was used as the experimental dataset.

### 3.1. Dataset

The UP-Fall dataset [19], a large motion trajectory dataset mainly used for fall detection, contains 11 activities. Three trials were performed for each activity obtained from 17 subjects (9 males and 8 females) performing six simple ADL activities and five different types of human falls. As shown in Table 1, these 17 healthy young subjects ranged in age from 18 to 24 years, had a mean height of 166 cm and a mean weight of 66.82 kg.

As shown in Table 2, all five human falls (falling forward using the hands, falling forward using the knees, falling backwards, falling sideways and falling from sitting in a chair) were performed over 10 s. Except for the sampling time of 30 s for jumping and 10 s for picking up an object, an activity easily mistaken as falling, the other four of the six simple ADLs (walking, standing, sitting, picking up an object, jumping and lying) were all conducted over 60 s.

In contrast to other existing datasets, the UP-Fall dataset contained measurement data from five sensors located at different parts of the body. These motion data included 3-axis accelerometer and 3-axis gyroscope data captured simultaneously by five Bluetooth sensor nodes, all with a sampling frequency of approximately 18 Hz.

**Figure 1:**
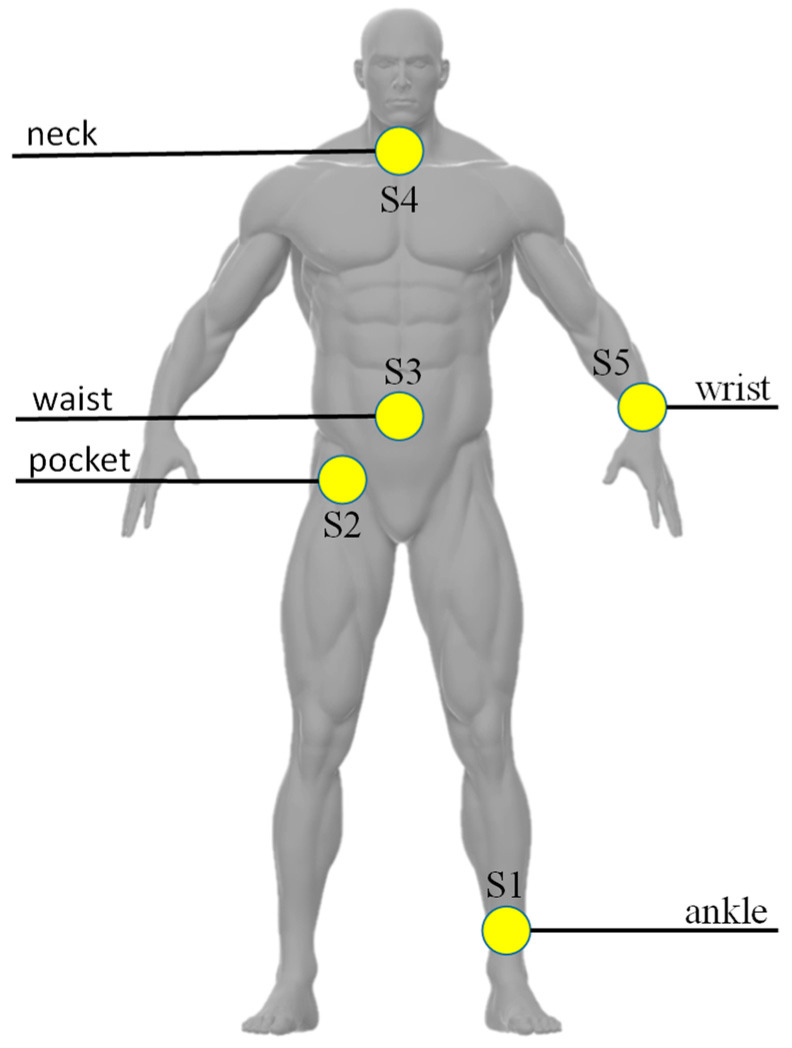
Distribution of IMU sensors located on the human body in the UP-FALL dataset.

When the data of all sensors were preprocessed, the skeleton model construction for fall detection was performed based on the natural connection relationship of the human body joints corresponding to the five sensors. Then the skeleton sequence was constructed as a spatial-temporal graph and input into the ST-GCN fall-detection model. As shown in Figure 1, five wearable sensors were used to collect raw data from the 3-axis accelerometer and 3-axis gyroscope in UP-Fall dataset. These wearable sensors were worn on the left ankle (S1), at the right pocket of the pants (S2), at the middle of the waist (S3), under the neck (S4) and on the left wrist (S5).

### 3.2. ST-GCN-Based Fall Detection

In this part, the overall workflow of the ST-GCN fall-detection method is presented in detail: the multiple inertial sensors data were windowed to build the spatial-temporal graph; then, the data were used to train the ST-GCN model; finally, the model was used to predict falls and ADLs in the testing experiment. The graph neural networks included in our proposed model are introduced. The establishment process of the ST-GCN-based fall-detection model is described.

#### 3.2.1. Workflow of Fall Detection

**Figure 2:**
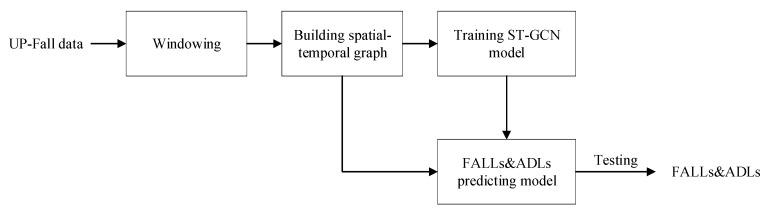
Overall workflow of ST-GCN fall-detection system.

The main workflow of fall detection is shown in Figure 2: the 3-axis acceleration and 3-axis angular velocity data collected by five inertial sensors in the UP-Fall dataset were used to build the fall-detection model. Firstly, the skeleton model of fall detection was established based on the natural connection between the human joints where the IMUs were located; secondly, the raw data were windowed using different window sizes: (a) one-second, (b) two-second, (c) three-second; thirdly, the appropriate ST-GCN model training parameters and hyperparameters were analyzed and determined, and the experimental results in different window sizes were discussed; finally, the fall-detection model based on ST-GCN was established, and the experimental results of ST-GCN algorithm were compared with those of seven other algorithms.

#### 3.2.2. Graph and Graph Neural Networks

A graph is a data structure consisting of a finite non-empty set of vertices and a set of edges between vertices, denoted as G(V, E), where G represents a graph, V is the set of vertices of the graph G, and E is the set of edges of the graph G. Generally speaking, a graph is considered as an abstract network of vertices, in which vertices can be connected to each other by edges, indicating that vertices are related. As non-Euclidean data, graph data does not have translation invariance, and its complex spatial structure poses a great challenge to existing ML and DL algorithms [26].

A graph neural network (GCN) is a hot area of machine learning research. The graph convolutional network is one of the graph neural networks, which has been successfully applied in traffic network analysis and text classification [27].

In the field of image processing, the ordinary convolution operation uses a number of fixed-size convolution kernels (filters) to scan the input image. Near the central pixel of each image, a pixel matrix with the same size as the weight matrix is extracted. This pixel matrix is used to produce the inner product with the convolution kernel to obtain the convolution output value of the central pixel. The neighborhood pixels can be defined as a neighborhood of the central pixel in the pixel matrix. When the convolution operation on an image is extended to an arbitrary graph structure, the neighborhood of any node can be defined with a series of weight matrices. This is the basic idea of graph convolutional networks.

However, unlike images, the number of nodes in the neighborhood of each node is not fixed if the neighborhood is defined using an adjacency matrix on an ordinary graph structure. This makes it difficult to determine the dimensionality of the convolution kernel to be used and how to arrange the order of the inner product operations of the weight matrix with the nodes in the neighborhood. In GCN, the inner product is computed using the same vector with the feature vectors on all the nodes in the neighborhood and the calculated results are averaged. This allows the dimensionality of the convolution kernel to be determined as a fixed length and the order of the nodes in the neighborhood does not need to be considered. This design allows GCN to be used on graphs with an arbitrary connectivity relationship.

#### 3.2.3. ST-GCN-Based Fall-Detection Algorithm

**Figure 3:**
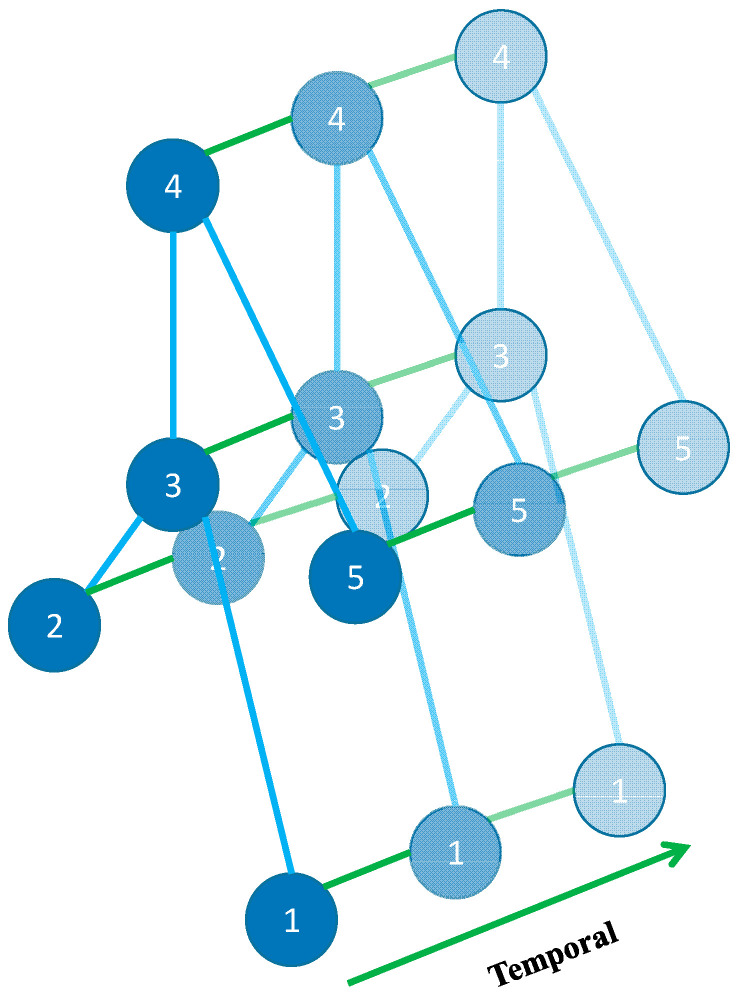
Spatial-temporal graph of the skeleton sequence built for fall detection.

In this paper, firstly the skeleton model for fall detection was established based on the natural connection between the human joints where IMUs are located in the UP-Fall dataset, and then the interframe connection of the nodes of the skeleton model was completed on the time axis, and finally the spatial-temporal graph of the skeleton sequence was obtained. As shown in Figure 3, the blue circular nodes represent the human body joints. The intrabody connection is defined based on the natural connection of the human joints. There are two types of edges, spatial edges, which are built on the naturally connected nodes of the human skeleton in each frame, and temporal edges, which connect the same nodes in two consecutive frames. Many layers in the spatial-temporal graph convolutional network are also built in this way, which integrate the motion information in the temporal and spatial domains.

In order to analyze the information about the human action of falling and ADLs in a spatial-temporal graph, the ST-GCN model abstracts the skeletal structure of the human body as a spatial graph, in which the vertexes are joints and the edges represent the natural connections of joints, and then constructs a graph network utilizing spatial-temporal convolutional blocks to perform feature extraction at the spatial and temporal scale, respectively. This method automatically captures the spatial and temporal dynamics of joints, which is one advantage of the graph convolutional network. However, as mentioned before, the skeleton is in the form of a graph rather than a 2D or 3D grid, which makes it difficult for models such as convolutional networks to be used. Recently, GCN, which generalize CNN to arbitrarily structured graphs, have received increasing attention and have been successfully adopted in many applications.

**Figure 4:**
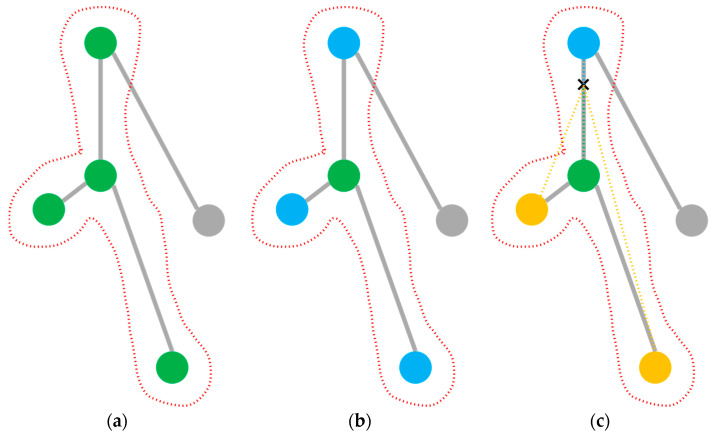
Three kinds of partition strategies. (**a**) Uni-labeling; (**b**) distance; (**c**) spatial configuration.

Since pixel points are tightly connected in an image, a traversal sequence naturally exists, such that convolutional kernels can traverse all pixel points on an image by cycling through them in a top-down, left-to-right order and be weighted with the central pixel point and its neighbors to obtain new image features. However, for graphs, there is no naturally existing order for traversal. Therefore, in order to be able to traverse the neighboring nodes, an adjacency matrix needs to be constructed. However, for a large graph structure, it is impossible to add all the node features directly. To partition the neighboring nodes in the graph structure, the ST-GCN [25] codes each neighboring node with a serial number, and the neighboring nodes with the same serial number are regarded as a neighboring subset, which also forms multiple adjacency matrices. Therefore, three partition strategies are proposed:

(a) Uni-labeling partitioning. All nodes in their central node neighborhood have the same label. As shown in Figure 4a, all green nodes in the neighborhood are all with the same label.
(b) Distance partitioning. By the distance between a node and the target node, a graph (such as a local skeleton graph of the human body composed of nodes) is divided into two parts. As shown in Figure 4b, the green nodes are nodes with distance of 0, that is, the nodes themselves, and the blue nodes are neighboring nodes with a distance of 1, that is, the nodes directly connected to the root node.
(c) Spatial configuration partitioning. As shown in Figure 4c, the distance from the root node (green) to the center of gravity of the skeleton (black cross) is taken as the baseline, and the nodes with a shorter distance to the center of gravity than the baseline are marked as centripetal nodes (blue), while the centrifugal nodes (yellow) have a longer distance than the baseline.

It should be noted that in addition to the graph-based spatial structure method mentioned above, there is also a spectral analysis-based construction method for applying neural networks on graphs. The graph-based spatial configuration was applied in this paper.

**Figure 5:**
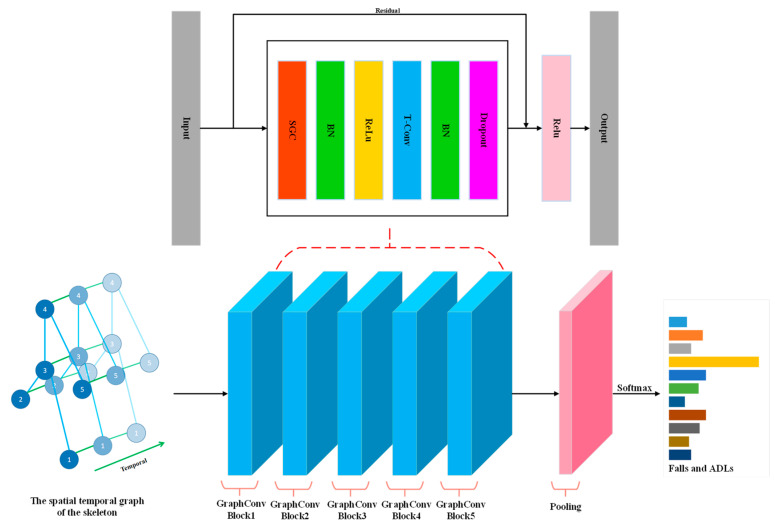
Illustration of the network structure of ST-GCN fall-detection model.

The overall structure of the network used in this paper is shown in Figure 5. The spatial-temporal graph is first input to several identical spatial-temporal blocks, all of which are composed of a spatial graph convolution (SGC) layer, two BN layers, a Relu layer, a temporal convolution (T-Conv) layer, and a dropout layer. The SGC layer is used to extract spatial static features from the spatial graph abstracted from the skeletal structure of the human body and the BN layer is constructed to normalize the data. The normalized data is fed to the ReLu layer for non-linear activation. The T-Conv layer is constructed to extract temporal dynamic features of falls and ADLs. The dropout layer is used to avoid model overfitting, and the residual connection is applied to guarantee the stability of model training. There are 5 basic spatial-temporal blocks in the model, with output channels of 32, 32, 32, 32, and 32. After that, the output tensor is fed into a global average pooling layer to obtain a feature vector for each sample of falls and ADLs. Finally, the vectors are passed into the output layer with a softmax function to obtain the prediction of falls and ADLs.

## 4. Experiments

In this section, we present the experimental settings and the metrics used to evaluate the performance of the fall-detection algorithms. In addition, the results of three types of experiments are shown in detail.

### 4.1. Experimental Setup

In this paper, the grid search strategy was used to determine the optimal hyperparameters of the fall-detection model; then, experiments with different window sizes were conducted to explore the effect of window size on model performance; finally, the fall-detection model of ST-GCN was established and the results of ST-GCN were compared with those of MLP, CNN, RNN, LSTM, TCN (temporal convolutional network) [28], TST (time series transformer) [29], and MiniRocket [30] to demonstrate the superiority of the ST-GCN algorithm for fall detection.

In this paper, the training and testing platform of the fall-detection model was built on a Windows 10 system, mainly implemented using Python and PyTorch. Table 3 shows the specific configuration information of the experimental environment.

For the experiments, the performance of fall-detection algorithms was evaluated using four metrics: accuracy, precision, recall, and F_1_-score, as shown in Equations (1)–(4); where *TP* and *TN* are the true positives and true negatives, and *FP* and *FN* are the false positives and false negatives.

(1) $$Accuracy=\frac{TP+TN}{TP+FN+TN+FP}\times 100\%\;$$

(2) $$Precision=\frac{TN}{TN+FP}\times 100\%\;$$

(3) $$Recall=\frac{TP}{TP+FN}\times 100\%\;$$

(4) $$F_{1}-Score=\frac{2\times Precious\times Recall}{Precious+Recall}\times 100\%\;$$

In this paper, the training parameters of ST-GCN and the other seven algorithms were first determined, as shown in Table 4.

In all experiments, the following strategy was used: 80% of the total data in the experimental dataset was used for training, 10% for validation, and 10% for testing, and the cross-entropy error function was used as the loss function for backpropagation. All models were trained for 1000 epochs, and the learning rate was set to 0.0001. Ten rounds of cross-validation were performed using different random partitions carried out by samples over each of the selected ML and DL methods. The results of ten rounds of experiments were averaged as the final results using 10-fold cross-validation method. All the fall-detection algorithms were tested using three different window sizes: (a) one-second, (b) two-second and (c) three-second. An overlap of 50% was considered in all the cases.

The partition strategies of convolutional operations (PS) and the maximum distance of the connection between neighboring nodes and the central node (MD) are very important for the ST-GCN model. In order to analyze the effects of PS and MD on the performance of the ST-GCN fall-detection model and find the optimal PS and MD, the grid-search experiment was conducted.

Table 5 shows the range of these two hyperparameters. The partitioning strategies of Uni-labeling, distance and spatial configuration were used respectively, and MD was set to 1 or 2. In the grid-search experiment, the window size was set to 2 s and the size of overlap was set to 1 s.

### 4.2. Experimental Results

#### 4.2.1. Determination of Model Parameters

As shown in Table 6, changing the PS and MD parameters had certain effects on the performance of the ST-GCN model with the other parameters unchanged. The ST-GCN model achieved the highest accuracy of 98.05% when the PS was spatial configuration and MD was 1.

Therefore, the PS and MD were set to spatial configuration and 1 in all subsequent experiments, respectively.

#### 4.2.2. Experimental Results of Different Window Sizes

The influence of window size on classification performance of ST-GCN was tested when the other parameters were unchanged.

In this paper, three sets of window-size experiments were conducted, and the window sizes were set to 1.0 s, 2.0 s, and 3.0 s.

Table 7 shows the performance comparison of the ST-GCN model using different window sizes in the four evaluation criteria. The ST-GCN model using the window size of 2.0 s almost achieved the best results according to all the criteria. The accuracy, precision, recall, and F_1_-score were 98.05%, 85.02%, 93.46%, and 88.30%, respectively. When compared with the results of 1 s window size, the ST-GCN model using the window size of 2.0 s improved the accuracy by 0.21%, precision by 1.75%, recall by 6.99%, and F_1_-score by 3.88%. When compared with the results of the 3 s window size, the ST-GCN model using the window size of 2.0 s improved the accuracy by 0.77%, precision by 6.97%, and F_1_-score by 4.73%. Of the three sets of experimental results, the results of the 3 s window size had the worst classification performance with the lowest accuracy, precision, and F_1_-score of 97.28%, 78.05%, and 83.57%, respectively; while only in terms of the recall, the experimental result with window size of 3.0 s was slightly higher than 2.0 s by 0.12%.

In summary, the overall recognition performance of the ST-GCN algorithm was best when the window size was set to 2.0 s and the overlap was set to 1.0 s.

#### 4.2.3. Experimental Results of Different Algorithms

In this section, the fall-detection method based on ST-GCN was compared with seven algorithms of MLP, CNN, RNN, LSTM, TCN, TST, and MiniRocket.

Table 8 shows the performance comparison between different fall-detection models in the four evaluation criteria. ST-GCN outperformed the other algorithms according to all the criteria. The ST-GCN model using the window size of 2.0 s achieved the highest accuracy of 98.05%, precision of 85.02%, and F_1_-score of 88.30%. Furthermore, the ST-GCN model using the window size of 3.0 s achieved the highest recall of 93.58%. The accuracy of TST ranked second among these models using a 2 s window size, at 97.57%. The accuracy of RNN was ranked last among these models using a 2 s window size, at 88.68%. The ST-GCN model improved by 0.48% in accuracy, 3.21% in precision, 14.16% in recall and 8.62% in F_1_-score compared with TST, and improved by 9.37% in accuracy, 28.91% in precision, 29.86% in recall and 30.72% in F_1_-score compared with RNN. When compared with the MLP algorithm [19], the ST-GCN model using the window size of 2.0 s improved the accuracy by 1.33%, precision by 11.27%, recall by 24.59% and F_1_-score by 17.95%. The improvement of the proposed ST-GCN model for fall detection over the other selected models in each evaluation criterion can also be observed in Table 8.

## 5. Discussion

In this section, we analyze the effects of partition strategies and different window sizes on ST-GCN performance; then, the performance of different models is compared, and the characteristics of these fall-detection methods are discussed.

### 5.1. Effects of Partition Strategies on ST-GCN Performance

Different partition strategies have different effects on the performance of the ST-GCN fall-detection model. Whether MD = 1 or MD = 2, the recognition accuracy of uni-labeling partition strategy was lower than the other two multi-subset partition strategies. Mainly because uni-labeling divides different nodes in the neighborhood using the same weight vector, which is equivalent to simply averaging the feature vectors of all nodes in the neighborhood before the convolution operation, so that differential properties of the skeleton between joints cannot be modeled. Therefore, the recognition performance of ST-GCN based on uni-labeling partition strategy was not particularly satisfactory. In contrast, with the multi-subset partition strategy, the nodes in the neighborhood are divided into two or three subsets, and then there are different weight vectors, which can learn the features of different nodes more discriminately and can model the local differential properties between skeleton joints (e.g., the relative translations between joints).

The motions of human body parts can be broadly classified into concentric and eccentric motions. In this experiment, the spatial configuration achieved the highest recognition accuracy, mainly because this partition strategy can better characterize the stationary, centripetal and centrifugal motions of the joints of the human skeleton and can give more attention to the extremity joints. Generally, the closer you are to the center of gravity, the smaller the amplitude of motion is, and the farther away you are from the center of gravity, the larger the amplitude of motion is.

ST-GCN [25] showed that the multi-subset partition strategy was usually better than the uni-labeling partition strategy, and using the spatial configuration in the multi-subset partition strategy led to better performance of the ST-GCN model. As shown in Table 6, the experimental results of fall detection in this paper also further validate this important conclusion.

### 5.2. Effects of Different Window Sizes on ST-GCN Performance

When the raw data are divided into windows, different window sizes affect the classification performance of the ST-GCN model. As shown in Table 7, it could be concluded that the overall classification performance of ST-GCN for falls and ADLs was best when the window size was 2.0 s and the overlap was 1.0 s, in which accuracy, precision, recall and F_1_-score were 98.05%, 85.02%, 93.46%, and 88.30%, respectively.

**Figure 6:**
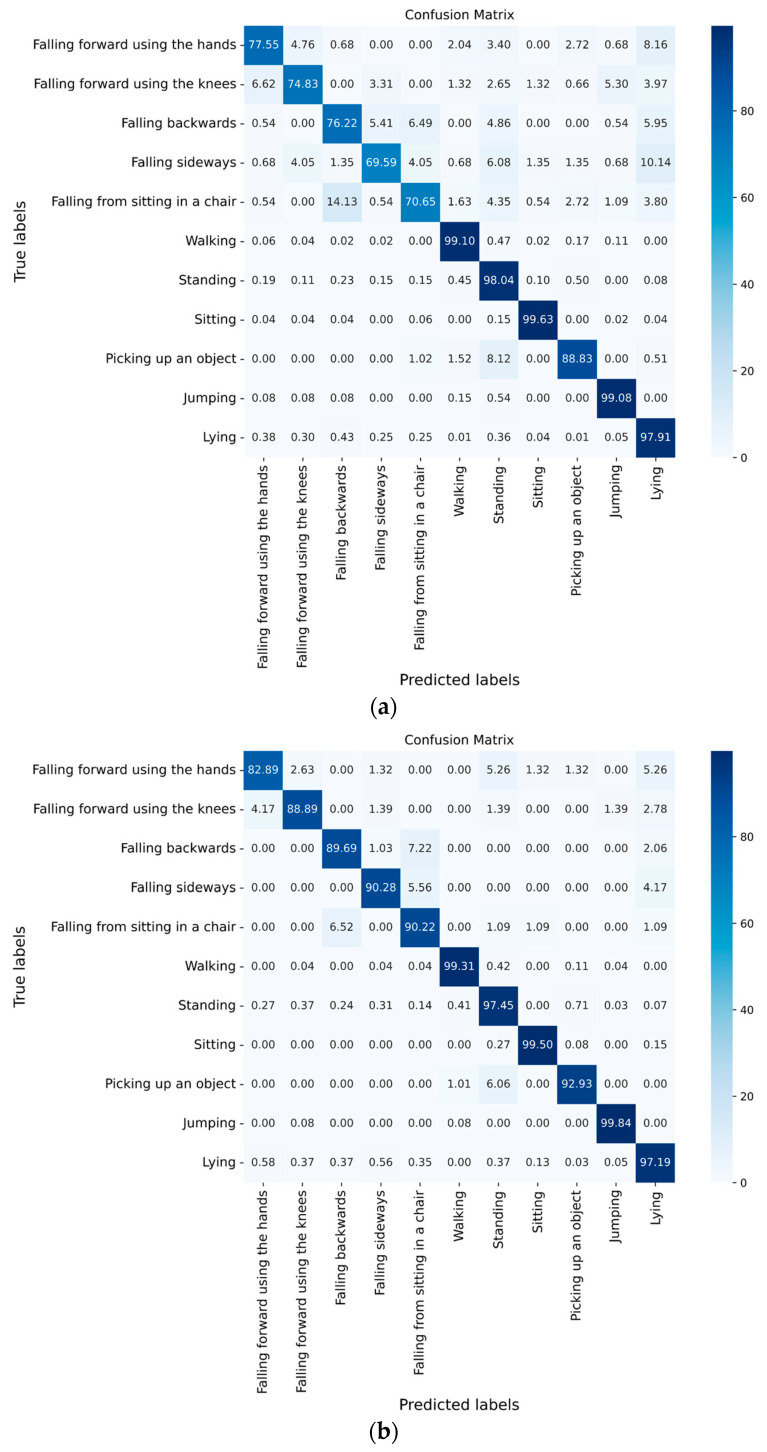

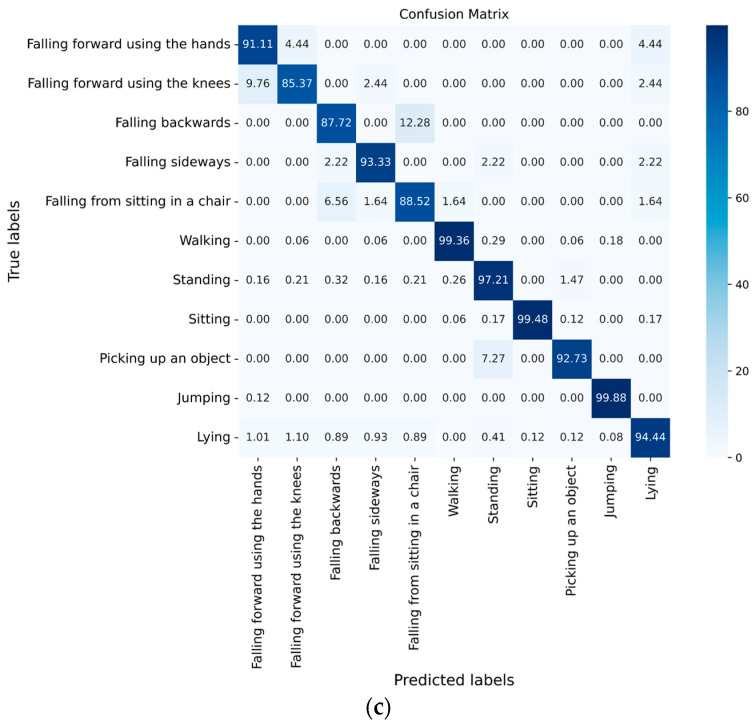
Confusion matrix of ST-GCN algorithm with different window sizes. (**a**) Window size = 1.0 s, overlap = 0.5 s. (**b**) Window size = 2.0 s, overlap = 1.0 s. (**c**) Window size = 3.0 s, overlap = 1.5 s.

As shown in Figure 6, in order to further analyze the influence of different window sizes on fall and ADL recognition, each confusion matrix with the window size of 1.0 s, 2.0 s, and 3.0 s was calculated, respectively.

As shown in Figure 6a–c, in the experimental results of the five types of falls, the recognition accuracy with a window size of 2.0 s of falling forward using the hands, falling forward using the knees, falling backwards, falling sideways, and falling from sitting in a chair was 5.34%, 14.06%, 13.47%, 20.69% and 19.57% higher, respectively, than that with a window size of 1.0 s. The recognition accuracy of falling forward using the knees, falling backwards and falling from sitting in a chair was 3.52%, 1.97%, and 1.70% higher, respectively, than that with a window size of 3.0 s.

In the experimental results of six ADLs, the recognition accuracy of walking, picking up an object and jumping with a window size of 2.0 s was 0.21%, 4.10% and 0.76% higher, respectively, than that with a window size of 1.0 s. The accuracy of standing, sitting, picking up an object and lying with a window size of 2.0 s was 0.24%, 0.02%, 0.2% and 2.75% higher than that with a window size of 3.0 s.

The recognition accuracy of the six ADLs was slightly higher than that of the five types of falls. One of the reasons may be the data imbalance of the UP-Fall dataset: the sampling time of ADLs was mostly 60 s, while the sampling time of each type of fall was 10 s. In addition, the data of ADLs such as walking, standing, jumping and lying were not changing too much or changing periodically; while during a 10 s sampling, all falls were performed only once, and a fall process only accounted for approximately 1/5 of the duration of a sample. Therefore, the sample size of falls was smaller compared with ADLs.

In the process of changing the window size, the five ADLs of walking, standing, sitting, jumping and lying kept high accuracy. Except for the accuracy of lying in the experiments with window size of 3.0 s, which was only 94.44%, the rest were all above 97%. The results showed that ADLs were slightly influenced by the window size.

In contrast, the five types of falls were more influenced by window size. The lowest accuracy was only 69.59% when the window size was 1.0 s; while the lowest accuracy with the window size of 2.0 s and 3.0 s was 82.89% and 85.37%, respectively. So, the results showed that the overall recognition accuracy of falls improved as the window size was increased within a certain range. One of the reasons may be that more information about a fall’s process can be included in a window when the window size is increased appropriately.

In summary, the highest overall accuracy was achieved when the window size was 2.0 s, and most of the falls and ADLs ranked high in accuracy, improving false recognition.

### 5.3. Performance Comparison of Different Algorithms

In order to verify the effectiveness and robustness of the proposed ST-GCN-based fall-detection algorithm, the experimental results of ST-GCN and seven other algorithms were compared and analyzed. In the three sets of experiments with window size of 1.0, 2.0 s and 3.0 s, ST-GCN performed the best of all the algorithms, with an accuracy of 97.84%, 98.05% and 97.28%, respectively.

To further analyze the performance of each algorithm, the F_1_-score of each action was calculated.

**Figure 7:**
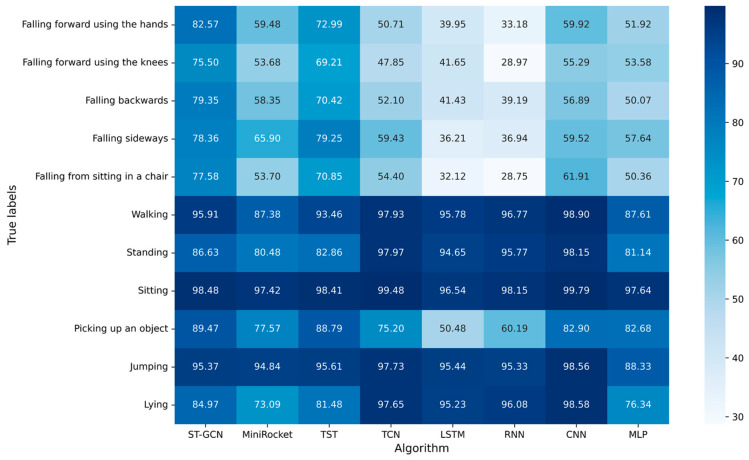
F_1_-score of each algorithm for all kinds of actions with window size of 1 s.

The experimental results with window size of 1.0 s are shown in Figure 7. The F_1_-score of ST-GCN ranked first in the five categories: falling forward using the hands, falling forward using the knees, falling backwards, falling from sitting in a chair and picking up an object, with 82.57%, 75.50%, 79.35%, 77.58%, and 89.47%, respectively. The F_1_-score of ST-GCN for falling sideways ranked second, with 78.36%.

**Figure 8:**
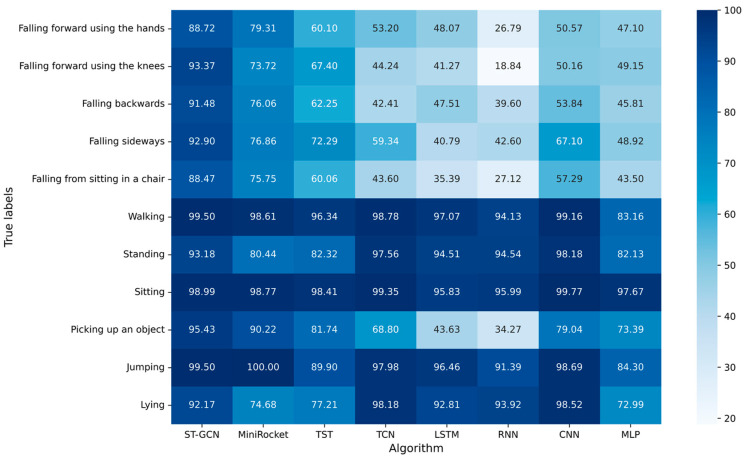
F_1_-score of each algorithm for all kinds of actions with window size of 2 s.

The experimental results with window size of 2.0 s are shown in Figure 8. The F_1_-score of ST-GCN ranked first in all falls: falling forward using the hands, falling forward using the knees, falling backwards, falling sideways and falling from sitting in a chair, with 88.72%, 93.37%, 91.48%, 92.90%, and 88.47%, respectively. The F_1_-score of ST-GCN also ranked first in the two ADLs: walking and picking up an object, with 99.50% and 95.43%, respectively. The F_1_-score of ST-GCN for jumping ranked second, with 99.50%. The F_1_-score of ST-GCN for sitting ranked third, with 98.99%.

**Figure 9:**
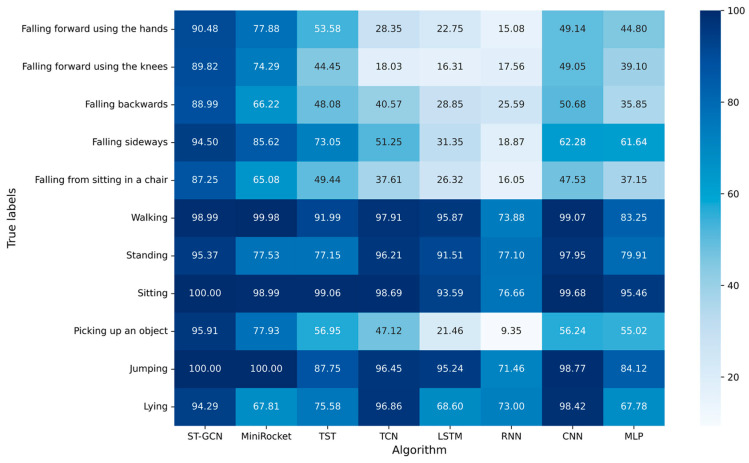
F_1_-score of each algorithm for all kinds of actions with window size of 3 s.

The experimental results with window size of 3.0 s are shown in Figure 9. The F_1_-score of ST-GCN ranked first in all falls, with 90.48%, 89.82%, 88.99%, 94.50%, and 87.25%, respectively. The F_1_-score of ST-GCN also ranked first in the three ADLs: sitting, picking up an object and jumping, with 100.00%, 95.91%, and 100.00%, respectively. The F_1_-score of ST-GCN for walking and lying ranked third, with 98.99% and 94.29%.

The ST-GCN model performed better than the other seven algorithms for five falls and picking up an object in the experimental results with window sizes of 2.0 s and 3.0 s. The results indicated that ST-GCN had higher accuracy and stability for both fall and ADL recognition compared to the other seven algorithms. Moreover, the proposed fall-detection method based on ST-GCN could reduce the false positives and false negatives.

The skeleton-based fall-detection model using ST-GCN achieved the best prediction performance in most experiments. Especially, in the five types of fall predictions, the performance differences between the ST-GCN model and some of the other models were significant, showing the proposed fall-detection model in this paper can better learn the dynamic spatial-temporal patterns of falling data.

As shown in Table 9, we compared the computational requirements of the eight fall-detection models using different window sizes in terms of the prediction time required for a single window. In general, these models can be sorted as CNN, ST-GCN, TCN, LSTM, RNN, MLP, TST, and MiniRocket in increasing order. The results show that ST-GCN can meet the real-time requirements of the fall-detection task.

Most of the ML and DL algorithms for time series (such as RNN, LSTM, TCN) mainly focus on temporal features and their abilities to learn spatial features are relatively weak. However, except for the temporal features, the data of falls and ADLs also have complex spatial patterns. In addition, recursive networks need iterative training, which will gradually introduce error accumulation. Therefore, the RNN-based networks used to capture the temporal correlations in the falling dataset performed worse than the ST-GCN model proposed in this paper. Compared with RNN, LSTM can better deal with long-term dependencies and mine the change rules of falls in historical data; TCN can allow parallel computation of outputs, which is more efficient than RNN but they still ignore the spatial patterns embedded in multi-inertial sensor data. In terms of TST, it also supports parallelism and has stronger long-term dependence modeling capability, but it is insensitive to local information and is susceptible to outliers. The CNN-based approaches, which transform the multisensor signals into grid-structured data or sequence-structured data as inputs, may not appropriately model the spatial dependencies in multisensor signals. MLP [19] needs to extract features manually, and the quality of features has a great influence on classification performance.

These deep learning algorithms seldom explicitly consider the spatial connectivity and graphical structure between joints. Therefore, they are relatively limited in understanding the behavior expressed by body movements, which leads to their overall worse performance than ST-GCN in human fall-detection problems.

ST-GCN applies graph neural networks to explicitly model the natural connections between the joints of the human body in space compared to traditional deep learning algorithms dealing with time series. The spatial convolution models the skeletal structure of the body as a static graph, making full use of the spatial structure information. To solve the inherent defects of recurrent networks, ST-GCN adopts a fully convolutional structure in the temporal axis. ST-GCN can automatically extract spatial-temporal motion patterns and realize the prediction of human falls and ADLs more accurately compared with other methods. ST-GCN with flexibility and scalability achieves faster training, easier transformation, and fewer parameters.

At the same time, a new partition strategy that goes beyond the average idea of the original GCN was proposed for ST-GCN, and the neighborhood set according to the partition strategy of spatial configuration was further divided, which enhanced the performance of the ST-GCN model for fall detection.

In summary, this paper proposed a new fall-detection method, which utilized the ST-GCN to make full use of human skeleton information and mine the spatial-temporal pattern of falls and ADLs among multiple inertial sensors. The results prove that this method can identify falls more accurately and reliably. On the UP-Fall dataset for fall detection, the proposed method achieved superior performance compared to previous methods.

## 6. Conclusions and Future Works

In this study, a fall-detection method based on the skeleton with multiple inertial sensors using ST-GCN was proposed. Firstly, the motion data of IMUs on the five joints of the human body were extracted from the UP-Fall dataset and windowed; secondly a skeleton model for fall detection was established according to the natural connection relationship between human joints; then, the spatial graph convolution layer and the temporal convolution layer were used to extract the dynamic features of falls and ADLs at the spatial and temporal scales, respectively, which more comprehensively mined the spatial and temporal patterns of human falls and ADLs. Finally, a fall-detection model based on ST-GCN and the skeleton was established; the influence of the two hyperparameters on the performance of the model was discussed and the experimental results were compared with those of seven other algorithms. The experimental results showed that the ST-GCN model with better accuracy and robustness outperformed the other seven algorithms. This study provides a new method for IMU-based fall detection.

The method proposed in this paper directly used the natural connections of human joints to build a human skeleton model for fall detection, but did not tap into the potential topological relationships of unnatural connections from the perspective of actual motion characteristics. For example, hands and feet are not naturally connected, but there are strong motion correlations during movement. In future work, attention mechanism can be used to self-learn certain unnatural connections of the skeleton during spatial information extraction to mine the non-topological relationships between human joints and improve the performance of fall detection.

## Figures and Tables

**Table 1:** Individual characteristics of the experimental subjects.

Characteristic	Range	Average	Standard Deviation	Median
Age	[18–24]	20.47	±1.66	20
Weight (kg)	[53–99]	66.82	±12.49	68
Height (cm)	[157–175]	166.47	±5.47	168

**Table 2:** Activities performed by subjects in the experimental dataset.

Category	Activity ID	Description	Duration(s)
Fall	1	Falling forward using the hands	10
	2	Falling forward using the knees	10
	3	Falling backwards	10
	4	Falling sideways	10
	5	Falling from sitting in a chair	10
ADL	6	Walking	60
	7	Standing	60
	8	Sitting	60
	9	Picking up an object	10
	10	Jumping	30
	11	Lying	60

**Table 3:** Experimental environment configuration.

Name	Configuration Information
OS	Windows10
Hardware	CPU:Intel i7-6700H Memory:16 GB Graphics card:RTX1060, 6 GB
Python library	Python3.8 Pytorch1.11.0 Skelarn0.23.1 Pandas1.0.5 Keras2.4.3

**Table 4:** Values of the training parameters of ST-GCN algorithm.

Parameter	Value
Minibatch_size	128
Learning_rate	0.0001
Max_epochs	1000
Patience	50/minibatches
Optimizer	Adam

**Table 5:** Range of the two model hyperparameters (PS and MD).

Parameter	Range
PS	[Uni-labeling, distance, spatial configuration]
MD	[1, 2]

**Table 6:** Accuracy of ST-GCN fall-detection model in the grid-search experiment (%).

	Uni-Labeling	Distance	Spatial Configuration
MD = 1	97.68	97.81	**98.05**
MD = 2	96.87	97.88	97.95

**Table 7:** Performance of ST-GCN fall-detection model using different window sizes: 1 s, 2 s, and 3 s.

Window (s)	Accuracy (%)	Precision (%)	Recall (%)	F_1_-Score (%)
1.0 s	97.84	83.27	86.47	84.42
2.0 s	**98.05**	**85.02**	93.46	**88.30**
3.0 s	97.28	78.05	**93.58**	83.57

**Table 8:** Performance of eight different fall-detection algorithms using different window sizes: 1 s, 2 s, and 3 s.

Model	Window (s)	Accuracy (%)	Precision (%)	Recall (%)	F_1_-Score (%)
MLP	1	97.32	77.47	72.58	74.45
	2	96.72	73.75	68.87	70.35
	3	96.40	74.93	65.56	67.74
CNN	1	97.45	78.84	79.57	78.80
	2	97.33	78.77	77.18	76.99
	3	97.13	75.72	74.11	73.52
RNN	1	94.95	64.79	69.61	66.49
	2	88.68	56.11	63.60	57.58
	3	72.25	45.28	53.82	44.85
LSTM	1	93.21	63.22	75.57	67.21
	2	91.11	59.92	71.87	63.06
	3	85.07	53.59	65.47	55.10
TCN	1	97.21	78.22	73.10	74.81
	2	96.81	74.98	70.72	71.76
	3	95.70	67.45	63.50	63.75
TST	1	97.77	82.10	78.42	79.88
	2	97.57	81.81	79.30	79.68
	3	97.45	80.07	75.38	76.42
MiniRocket	1	96.17	72.29	62.63	65.75
	2	96.19	71.45	62.87	65.39
	3	96.32	70.38	61.92	64.13
ST-GCN	1	97.84	83.27	86.47	84.42
	2	**98.05**	**85.02**	93.46	**88.30**
	3	97.28	78.05	**93.58**	83.57

**Table 9:** Prediction time per window of each fall-detection model using different window sizes: 1.0 s, 2.0 s, and 3.0 s.

Window Size (s)	MLP	CNN	RNN	LSTM	TCN	TST	MiniRocket	ST-GCN
	Prediction Time per Window (ms)							
**1.0**	1.11	0.10	0.26	0.31	0.33	11.21	16.82	0.16
**2.0**	1.50	0.07	0.29	0.28	0.23	13.32	26.13	0.13
**3.0**	0.54	0.07	0.35	0.26	0.21	12.70	36.74	0.11

## Data Availability

A publicly available dataset was analyzed in this study. This data can be found here: http://sites.google.com/up.edu.mx/har-up/.
